# Cross-sectional survey on impact of paediatric COVID-19 among Italian paediatricians: report from the SIAIP rhino-sinusitis and conjunctivitis committee

**DOI:** 10.1186/s13052-020-00906-4

**Published:** 2020-10-06

**Authors:** Lucia Diaferio, Giuseppe Fabio Parisi, Giulia Brindisi, Cristiana Indolfi, Giuseppe Marchese, Daniele Giovanni Ghiglioni, Anna Maria Zicari, Gian Luigi Marseglia, Michele Miraglia del Giudice

**Affiliations:** 1 Department of Pediatrics, Giovanni XXIII Hospital, University of Bari, 70126 Bari, Italy; 2 Department of Clinical and Experimental Medicine, University of Catania, Via Santa Sofia 78, 95123 Catania, Italy; 3 Pediatric Respiratory Unit, Department of Clinical and Experimental Medicine, University of Catania, Viale Carlo Azeglio Ciampi, 95121 Catania, Italy; 4 Department of Pediatrics, Allergology and Immunology Division, Sapienza University, Viale Regina Elena, 324 Rome, Italy; 5 Department of Woman, Child and Specialized Surgery, University of Campania “Luigi Vanvitelli”, Via L. De Crecchio 2, 80138 Naples, Italy; 6 Primary care pediatrician, Cedegolo, Brescia, Italy; 7 Fondazione IRCCS Ca’ Granda Ospedale Maggiore Policlinico di Milano, Via Francesco Sforza, 28, 20122 Milan, Italy; 8 Department of Pediatrics, Foundation IRCCS Policlinico San Matteo, University of Pavia, Viale Camillo Golgi 19, 27100 Pavia, Italy

**Keywords:** Children, Coronavirus, COVID-19, Allergy, Asthma, Upper airway, Survey, Paediatricians

## Abstract

**Background:**

There is ample evidence that COVID-19 is significantly less severe in children than in adults and asthma and allergy, the most common chronic disorders in children, are not included in the top 10 comorbidities related to COVID-19 fatalities. Nevertheless, concerns about asthma and allergy are still high.. In order to evaluate the impact of paediatric COVID-19 among Italian paediatricians, we sent a 20-questions anonymous internet-based survey to 250 Italian paediatricians with particular address to allergic symptoms and those affecting the upper airways.

**Methods:**

The questionnaire was conceived and pretested in April 2020, by a working group of experts of the Italian Paediatric Society for Allergy and Immunology (SIAIP), and structured into different sections of 20 categorized and multiple choice questions. The first part included questions about epidemiological data follows by a second part assessing the way to manage a suspected COVID-19 infection and personal experiences about that. The third part concerned questions about patients’ clinical characteristics and clinical manifestations.

The survey was emailed once between April and mid-May 2020.

**Results:**

A total 99 participants had participated in our survey and provided responses to our electronic questionnaire. The distribution of patients reported per month varies significantly according to the geographical area (*P* = 0.02). Data confirmed that in the North part of Italy the rate of patients referred is higher than in the rest of Italy. Almost all respondents (98%) reported caring for up to a maximum of 10 infected children and the last 2% more than twenty. Among these patients, according to the 75% of responders, a maximum rate of 20% were affected by allergic rhino-conjunctivitis and in particular in the North of Italy while in the Centre and in the South there was a higher incidence (*P* = 0.09). Almost the same applies for asthma, 83% of responders declared that up to a maximum of 20% of affected children were asthmatic, from 20 to 40% for the 13,5% of responders and from 40 to 60% for the last 3,5%. As for the allergic conjunctivitis also for asthma, we found a higher incidence in the Centre and in South than in the North (*P* = 0.03).

**Conclusions:**

This study is the first to provide a comprehensive review of COVID-19 knowledge and impact among paediatricians in Italy about allergic asthma and upper airway involvement. From our point of view, it provides important information clearly useful for improving a good practice.

## Background

In March 2020, the World Health Organization (WHO) declared the COVID-19 pandemic. The novel coronavirus, SARS-CoV-2, a top threat to global health, emerged in Wuhan (China) in December last year and rapidly spread worldwide [1].

Globally, at the end of August 2020 the total confirmed cases of COVID-19 have reached over 24,765,000 with over 837,000 deaths and daily data shows continuous increases in new COVID-19 cases [1].

In Italy, we have experienced serious outbreaks linked to the first cluster, in South Lombardy, with about 300,000 confirmed cases and more than 35,000 deaths [1].

Research shows that COVID-19 causes symptoms including fever, dry cough, dyspnoea, fatigue, lymphopenia and in more severe cases, severe acute respiratory syndrome (SARS) and even death [2–5].

Every age may be affected but childhood seems to be safeguarded by severe COVID-19, due to comorbidities associated with lethal COVID-19 infection (obesity, diabetes and chronic heart disease) [6].

It has been reported that asthma and allergy, the most common chronic disorders in children, are not included in the top 10 comorbidities associated with COVID-19 fatalities [7].

Nevertheless, there would seem that the concerns about asthma and the risk of disease and related outcomes are still high [8].

Actually, data on COVID-19 in Italian children are limited and almost certainly underestimated, since they are frequently asymptomatic or presenting mild or moderate infection, similar to common cold.

In order to evaluate the impact of paediatric COVID-19 among Italian paediatricians, we sent a 20-questions anonymous internet-based survey to 250 Italian paediatricians with particular address to allergic symptoms and those affecting the upper airways.

## Methods

The questionnaire was conceived and pretested in April 2020, by a working group of experts of the Italian Paediatric Society for Allergy and Immunology (SIAIP) based on their personal clinical experience and on the extensive review of most relevant international literature on COVID-19 infection searched on MEDLINE, EMBASE and SCOPUS.

The prior revised and confirmed paper version of the questionnaire was finally converted in a web-based survey with Google-Drive (Google Drive™,© 2012 Google Inc. all rights reserved), a free internet platform applied for the creation of internet-based survey forms which allows to have real-time digital archiving of collected data, real-time presentation of survey results, and simple download of all data of registered anonymised participants in Excel© format for statistical analysis.

The questionnaire was structured into different sections of 20 categorized and multiple choice questions. The first part included questions about epidemiological data follows by a second part assessing the way to manage a suspected COVID-19 infection and personal experiences about that. The third part concerned questions about patients’ clinical characteristics and clinical manifestations.

Finally, the last part focused on the knowledge in the field and educational priorities of participants.

The language of the questionnaire was the national one.

The reported time to complete the survey was approximately 10 min.

The survey was emailed once between April and mid-May 2020 to about 250 members of the Italian Paediatric Society for Allergy and Immunology (SIAIP). Participants were allowed to complete only a single survey, duplicate entries were avoided and responses were scrupulously monitored.

Informed consent was not obtained, given that the participation was voluntary. No financial incentive was offered.

The Ethics Committee of the University of Bari (Italy), was contacted and no special permission was deemed to be required because the study design satisfied the criteria of an activity audit.

Once the questionnaire results were obtained, they were statistically processed.

Answers were converted in different categorical variables. Differences in categorical variables were evaluated with Chi square and Fisher exact tests as appropriate.SAS® University Edition (Cary, NC: SAS Institute Inc) was used for all analyses. Data are expressed as percentage, *p* < 0.05 were considered statistically significant.

## Results

A total 99 participants had taken part in our survey and provided responses to our electronic questionnaire by May 15th, 2020. The characteristics of the survey participants are detailed in Table 1**.**

**Table 1:** Survey participants’ characteristics

Total of participants		99	
Sex (male *vs.* female)		44.9% *vs.* 55.1%	
Age (y/o):			
I. II. III. IV. V.	20-30 31-40 41-50 51-60 >60	I. II. III. IV. V.	1.0% 11.2% 9.2% 40.8% 37.8%
Types of paediatricians:			
I. II. III. IV. V. VI.	Primary care Paediatric hospital medicine Paediatric emergency medicine Specialised outpatient healthcare Paediatric critical care medicine Paediatric infectious disease	I. II. III. IV. V. VI.	60.0% 24.2% 6.3% 6.3% 2.1% 1.1%
Territorial subdivisions (South and Islands *vs.* Centre of Italy *vs.* North of Italy)		40.2% *vs.* 27.8% *vs.* 32.0%.	

Among responders, 52% practiced in a place where there is not a Children Hospital dedicated to COVID care. 86% of respondents reported that in a month referred to them up to 10 patients for suspected SARS-CoV-2 and up to 20 for the 11% (more than 20 just for the 3%), starting from February 2020 according the majority of them (86%). In particular, the distribution of patients reported per month varies significantly according to the geographical area (*P* = 0.02). Data showed that in the North part of Italy the rate of patients referred is higher than in the rest of Italy. Moreover, we found that only the infectious disease specialist reported that in a month referring more than 20 patients for suspected SARS-CoV-2 (*P* < 0.0001).

The diagnosis of COVID-19 is made once a month according to 34% of participants, once a week for 23% of participants, once in 2 months for 19%, once in 3 months for 10%, and once a day for 9% of participants. Almost all respondents (98%) reported they had in charge up to a maximum of 10 infecting children and the last 2% more than twenty. Among these patients, according to the 75% of responders, a maximum rate of 20% were affected by allergic rhino-conjunctivitis and in particular in the North of Italy while in the Centre and in the South there was a higher incidence (*P* = 0.09).

**Fig. 1:**
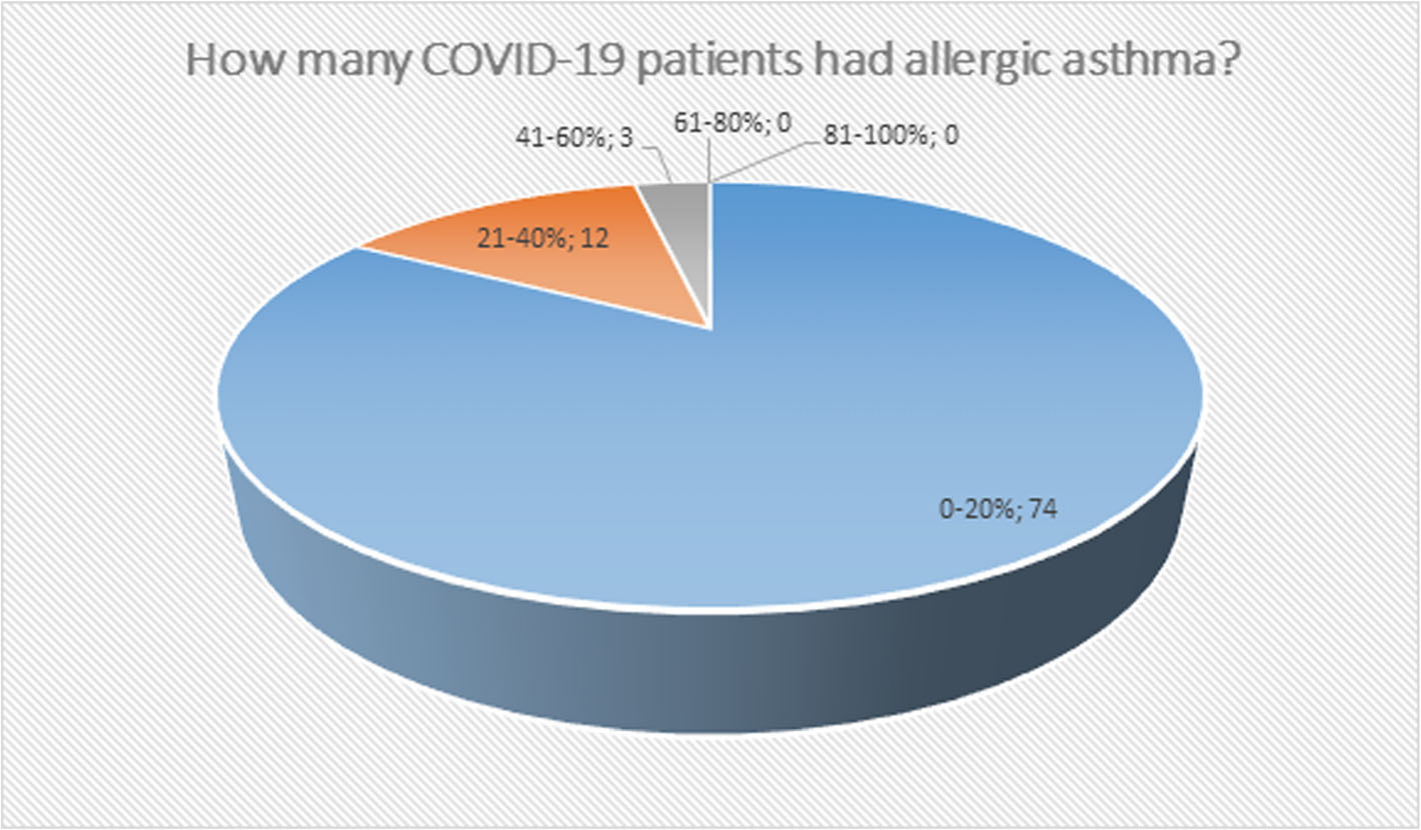
Answer to the question: How many COVID-19 patients had allergic asthma?

Almost the same applies for asthma, 83% of responders declared that up to a maximum of 20% of affected children were asthmatic, from 20 to 40% for the 13,5% of responders and from 40 to 60% for the last 3,5% **(**Fig. 1**)**. As for the allergic conjunctivitis also for asthma, we found a higher incidence in the Centre and in South than in the North (*P* = 0.03) (Table 2) .

**Table 2:**
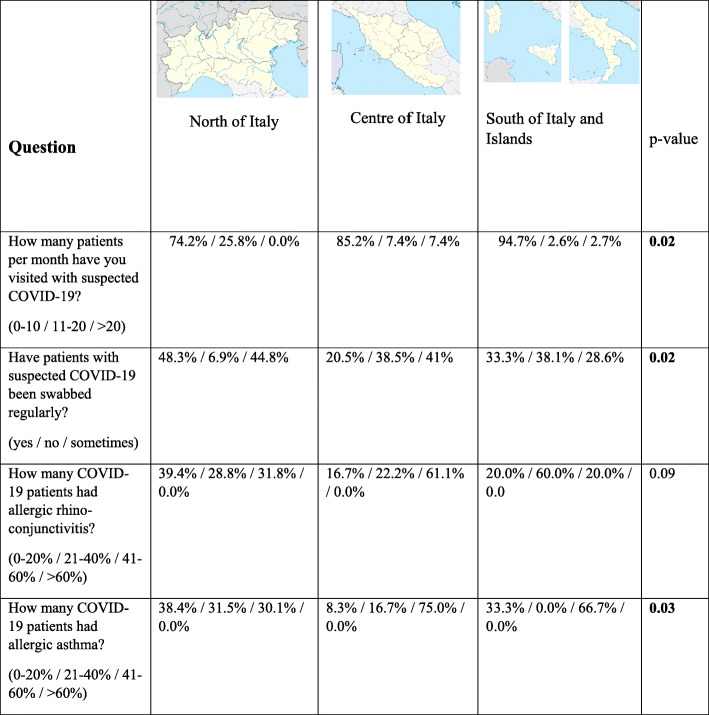
Evaluation of the responses to the questionnaire in the different macro-regions of Italy

On average, these children are ≤3 years old according to 24% of participants, from ≤4 to 6 years old for the 25% of responders as well from ≤7 to 10 years old, until 15 years old for the 21% and more than 16 years old for the last 5%.

Of the respondents, 90% agreed immediately isolation in a proper place and to alert the public health service system was the first step in case of a suspected infection, 13% declared to suggest just the isolation without any geographical differences. Eleven percent of respondents would refer out patients to the emergency department and the last 10% leading to an emergency call. However, 45% of participants clarified that confirmed cases of SARS-CoV-2 infection had nasopharyngeal and oropharyngeal swab sampling, 32% reported that it was not performed in suspected cases and not yet for 23% of responders. In particular, we found that the rate of not performed nasopharyngeal and oropharyngeal swab sampling is higher in the Centre and in the South than in the North (*P* = 0.02).

**Fig. 2:**
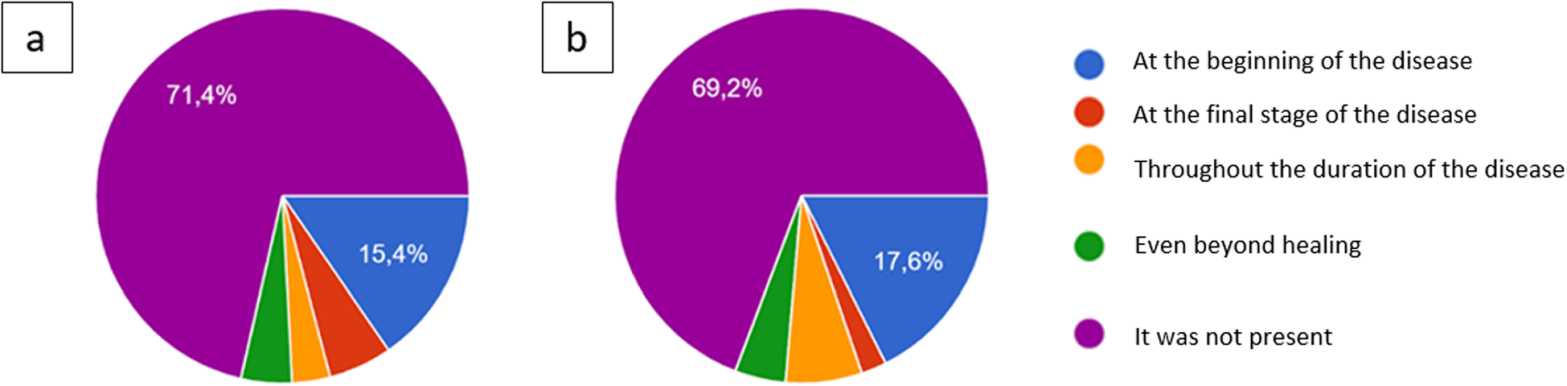
Answers to the questions: **a** Anosmia was one of the first symptoms reported in the literature. Among your patients, when did it occur? **b** Ageusia was one of the first symptoms reported in the literature. Among your patients, when did it occur?

Regarding signs and symptoms suggestive of SARS-CoV-2 infection, the majority of respondents (89%) recognized fever, cough (63%) and gastrointestinal disease (37%) as main symptoms. Interesting, olfactory and gustatory dysfunctions in children are rare **(**Fig. 2**).**

Finally, the majority of Italian paediatricians (85%) declared to have a good knowledge about COVID-19, however they’d all be interested to increase knowledge about the impact of COVID-19 on Italian children.

## Discussion

This cross-sectional survey provides information on the impact of COVID-19 among paediatricians.

. A good level of knowledge in the field is linked to a successful practice. So that, evaluating knowledge, attitude and practice among paediatricians is of considerable practical importance.

It should be noted that responders were allocated evenly among Italy in order to guaranteed information from all Italian regions. In addition, no significant difference has been identified with regard to management of a suspected COVID-19 case among Italy.

Regarding signs and symptoms suggestive of COVID-19, our results showed that in children, unlike adults, olfactory and gustatory dysfunctions are not prevalent. These findings are in line with a recent meta-analysis which included research performed in China (just one clinical case in Singapore) [9, 10].

Allergic rhino-conjunctivitis and asthma, according to our data, seem not to be a risk factor to developing more severe COVID-19. However, since the role of asthma in increasing the severity of COVID-19 is still unclear, it remains a great concern for patients and paediatricians.

The diagnosis and management of COVID-19 in children is still difficult due to the mild or moderate clinical course. Moreover, asymptomatic infections were not infrequent [8] with the risk of unconfirmed disease. This seems to be a frequent problem in daily clinical practice.

Nevertheless, our data showed that Italian children have good chances to be tested for SARS-CoV-2, indicating the importance of an accurate diagnosis, which will facilitate appropriate treatment options and preventive measures.

Our study shows some limitations. Although almost 100 participants completed our survey, only those with access to the Internet and only those with available email addresses were recruited. Other limitations are related to our pilot survey and include the use of a non-standardised questionnaire. However, to the best of our knowledge, standardised and validated surveys on this issue are not available. Some selection bias includes the recruitment methodology; those who felt more interested about COVID-19 may have been more inclined to complete the survey.

## Conclusions

This study is the first to provide a comprehensive review of COVID-19 knowledge and impact among paediatricians in Italy about allergic asthma and upper airway involvement. From our point of view, it provides important information clearly useful for improving a good practice. Our data confirmed that comorbidities as asthma or rhino-conjunctivitis cannot represent a risk factor for more severe COVID-19 disease. Moreover, symptoms such as anosmia and ageusia are rare in the paediatric population.

## Data Availability

The datasets used and/or analysed during the current study are available from the corresponding author on reasonable request.
